# To Lighten a Burden

**Published:** 1924-12

**Authors:** 


### TO LIGHTEN A BURDEN.

In an effort to help to liquidate the debt of £65,000 encumbering the Royal Northern Hospital (that is, the late Great Northern Central Hospital, with which is amalgamated the Royal Chest Hospital), a dinner was held at the Hotel Cecil, on November 13, when the guests were received by H.R.H. Prince Arthur of Connaught, supported by the Marquess of Northampton, the chairman.

THE SPEECHES.

The big banqueting hall was entirely filled, and music was provided by the band of the Scots Guards. The toasts were "Success to the Hospital," proposed by Prince Arthur, and replied to by the Marquess of Northampton, both of whom made earnest appeals for money on behalf of the hospital; "The Voluntary System," proposed by Sir Arthur Stanley, and replied to in a witty speech by the Rt. Hon. H. J. Tennant; "The Chairman," proposed by Sir Philip Sassoon, and seconded by Mr. Malcolm Donaldson, F.R.C.S., obstetric physician to the hospital. Lord Northampton was happily able to announce a generous offer made by Sir H. J. Williams, who for many years has represented the borough of Islington on the London County Council, to give £20,000 to the hospital as a memorial to his son who fell in the war, on condition that a similar sum is raised by January next. There was, in addition, an offer of £3,200 for the foundation of a maternity department, provided £5,000 for its endowment is received before the end of the year. Nor was this the end of the evening's good news. Mr. Gilbert Panter, the hospital secretary, was presently able to announce that gifts made at the dinner amounted to £6,654, of which the largest amount (£500) was given by Sir Philip Sassoon, treasurer of the hospital. Another offer of £500 was made by Sir John Latta, on condition that five similar amounts were forthcoming. So the happy ending to the dinner was that the hospital's coffers are now swelled by the sum of £6,654, with the prospect of a further £51,000, if all the amounts attached to conditional offers are subscribed.

